# Special Issue “Lignocellulosic Biomass”

**DOI:** 10.3390/molecules26051483

**Published:** 2021-03-09

**Authors:** Alejandro Rodríguez, Eduardo Espinosa

**Affiliations:** BioPrEn Group, Chemical Engineering Department, Universidad de Córdoba, 14014 Córdoba, Spain; a02esvie@uco.es

The use of lignocellulosic biomass as potential raw material for fractionation and transformation into high value-added products or energy is gathering the attention of scientists worldwide in seeking to achieve a green transition in our production systems. In this context, the aim of compiling recent advances in this field has motivated this Special Issue.

Lignocellulosic biomass is a valuable renewable and undervalued source of chemicals for use in the processing industry and can be used directly or indirectly for the production of platform molecules or bioproducts through chemical, physical, microbial, or enzymatic treatments and be used in sectors such as food, health, medicine, energy, materials, and the chemical industry. In maintaining this process, the scientific community plays a very important role in generating the basic knowledge that gives rise to technology and allows developments in the laboratory to be transferred to society. The integral valorization of lignocellulosic biomass is a fundamental pillar of sustainable development. Given the origin of this biomass, as well as its composition, lignocellulosic biomass is a vast resource.

In this Special Issue, twelve original research articles and three research reviews covering some of the most recent advances in lignocellulosic biomass fractionation and conversion for different applications are reported. Three articles deal with the production of biomass-derived biocomposites to replace oil-based products in different applications. Ehman et al. [1] reported on the use of bio-polyethylene (BioPE), sugarcane bagasse pulp, and two compatibilizers (fossil and bio-based) to produce biocomposite filaments for 3D printing. They show the influence of bagasse fiber fraction on the mechanical properties of the biocomposites as well as the reduction in CO_2_-equivalent emissions from replacing fossil compatibilizers with a bio-based compatibilizers in a cradle-to-gate life cycle analysis. Serra-Parareda et al. [2] studied the feasibility of incorporating barley straw fiber as reinforcement in a bio-based polyethylene to develop a fully bio-based and 100% recyclable material. They analyze the efficiency of barley fibers by the addition of anhydride maleic polyethylene as coupling agent to determine the flexural behavior of the material to determine the suitability of the material for several applications. Karagiannidis et al. [3] evaluated the use of micro-fibrillated cellulose (MFC) in waterborne adhesive systems applied in the manufacture of composite wood-based panels. They test the potential of improving the performance of wood-based panel types such as particleboard, waferboard, or randomly-oriented strand board and plywood, by the application of MFC and the substitution of conventional and non-renewable chemical compounds.

Five articles explore and develop the use of biomass-derived products as new industrial alternatives for different applications. Ortiz et al. [4] designed and prepared fully bio-based epoxy resins by combining epoxidized linseed oil, lignin, and bio-based diamine derived from fatty acid dimers. They showed that as the lignin content in the resin increases, the glass transition, the Young’s Modulus and the onset of thermal degradation increases. This correlation is non-linear, and the higher the percentage of lignin, the more pronounced the effect. All the components of the epoxy resin being commodity chemicals, the present system provides a realistic opportunity for the preparation of fully biorenewable resins at an industrial scale. Bascón-Villegas et al. [5] explored the use of horticultural plant residues (tomato, pepper, and eggplant) as new sources for lignocellulose nanofiber (LCNF) isolation using two different pretreatments, mechanical and TEMPO (2,2,6,6-Tetramethyl-piperidin-1-oxyl)-mediated oxidation, and followed by high-pressure homogenization. LCNF were added as reinforcing agent in recycled paperboard and compared with conventional mechanical beating. It showed that the addition of LCNF is a viable alternative to mechanical beating, achieving greater reinforcing effect and increasing the products’ life cycles. Szufa et al. [6] investigated the effect of biogas plant waste on the physiological activity, growth, and yield of Jerusalem artichoke and the energetic usefulness of the biomass obtained in this way after the torrefaction process. They achieved an increase in the calorific value process from 15.82 to 22.12 MJ kg^−1^ by this process. Nikolic et al. [7] investigated the potential of kraft lignin as a support material for the removal of hydrogen sulfide (H_2_S) from gaseous streams, such as biogas. The removal of H_2_S was enabled by copper ions that were previously adsorbed on kraft lignin. They reported a removal capacity of 2 mg H_2_S per gram of kraft lignin, and further studies in this technology being necessary to increase the viability of this technology. Michel et al. [8] reported the modification with β-cyclodextrin (βCD) of TEMPO-oxidized cellulose nanofibers (toCNF) samples with different carboxyl contents for biomedical applications. They reported covalent esterification binding between β-cyclodextrin and toCNF under acidic pH by freeze-drying, showing an interesting impact on the mechanical properties in the swollen state. This study is a step towards the production of mechanically tailored cryogels containing cyclodextrin, making them promising materials for the sustained delivery of active principal ingredients.

Three articles report novel advances in the use of biomass for the production of platform molecules and their conversion into high value-added products. Padilla-Rascón et al. [9] evaluated the conditions under which furfural concentration is maximized from a synthetic, single-phase, homogeneous xylose medium. They performed the experiments in a microwave reactor using FeCl_3_ and sulfuric acid as catalysts, showing the best operational conditions for a 57% furfural yield production at 210 °C, 0.5 min, and 0.05 M FeCl_3_. Oliva-Taravilla et al. [10] investigated the use of four biosurfactants, namely, horse-chestnut escin, *Pseudomonas aeruginosa* rhamnolipid, and saponins from red and white quinoa varieties, on the enzymatic saccharification of steam-pretreated spruce. They reported that the use of biosurfactants improved hydrolysis up to 24%, showing the potential of biosurfactants for enhancing the enzymatic hydrolysis of stem-pretreated softwood. Jiménez-Quero et al. [11] explored the optimization of solid-state fermentation (SSF) with lignocellulosic biomasses using *Aspergillus terreus* and *Aspergillus oryzae* to produced itaconic and fumaric acids. *A. oryzae* on corn cobs at specific conditions showed the best yield in acid production, achieving 0.05 mg of itaconic acid and 0.16 mg of fumaric acid per gram of biomass after 48 h in a large-scale fermentation process.

One of the articles present a novel protocol for the modification of dialdehyde cellulose (DAC) by silanization processing. Lucia et al. [12] presented a straightforward protocol, which meets common green chemistry principles, for the direct silanization of DAC, one of the most recently developed and studied cellulose derivatives, describing the direct silanization of DAC with (3-aminopropyl)triethoxysilane (APTES), through thermal treatment and freeze-drying.

Three review papers complete this Special Issue. The first one summarizes the evolution, year by year, of the development of the "lignin first" biorefinery approach [13]. A compact summary of achievements, future prospects, and remaining challenges is provided in this review. The second one outlines the possibility of applying known biorefinery processes to banana agro-industrial residues to generate high-value products from this residual biomass source [14]. It details information on the Central and Latin American context of this residue and the advantages of its use as raw material for the production of different biofuels, nanocellulose fibers, different bioplastics, and other high value products. The last review addresses the current knowledge on the electrochemical conversion of bio-based chemicals, particularly saccharides, to commodity chemicals [15]. Both oxidation and reduction pathways are shown with the most recent examples. Further recommendations are reported about the research needs, choice of electrocatalyst and electrolyte, as well as upscaling the technology.

Given the diversity of the contributions, it is evident that a multidisciplinary approach is needed to continue advancing the development of technologies and processes for the valorization of lignocellulosic biomass. There are still significant barriers to be overcome in order to achieve a complete transition of our production systems, from a petroleum-based economy to a bio-based economy. It is therefore expected that this field will be of particular relevance in the coming years. Finally, the guest editors would like to sincerely thank all the authors for their valuable contributions.
